# Genetic mapping and marker development for resistance of wheat against the root lesion nematode *Pratylenchus neglectus*

**DOI:** 10.1186/1471-2229-13-230

**Published:** 2013-12-31

**Authors:** Dimanthi V Jayatilake, Elise J Tucker, Harbans Bariana, Haydn Kuchel, James Edwards, Alan C McKay, Ken Chalmers, Diane E Mather

**Affiliations:** 1 School of Agriculture, Food and Wine, Waite Research Institute, The University of Adelaide, PMB 1, Glen Osmond, SA 5064, Australia; 2 Australian Centre for Plant Functional Genomics, Waite Research Institute, The University of Adelaide, PMB 1, Glen Osmond, SA 5064, Australia; 3 The University of Sydney Plant Breeding Institute – Cobbitty, PMB 4011, Narellan, NSW 2567, Australia; 4 Australian Grain Technologies, PMB 1, Glen Osmond, SA 5064, Australia; 5 South Australian Research and Development Institute, Plant Research Centre, 2b Hartley Grove, Urrbrae, SA 5064, Australia

**Keywords:** Root lesion nematode, *Pratylenchus neglectus*, Wheat, Molecular markers, *Rlnn1*, *Lr20*, *Sr15*, *Psy-A1*

## Abstract

**Background:**

The *Rlnn1* locus, which resides on chromosome 7A of bread wheat (*Triticum aestivum* L.) confers moderate resistance against the root lesion nematode *Pratylenchus neglectus*. Prior to this research, the exact linkage relationships of *Rlnn1* with other loci on chromosome 7A were not clear and there were no simple codominant markers available for selection of *Rlnn1* in wheat breeding. The objectives of the research reported here were to (1) develop an improved genetic map of the *Rlnn1* region of chromosome 7A and (2) develop molecular markers that could be used in marker-assisted selection to improve resistance of wheat against *P. neglectus.*

**Results:**

A large-effect quantitative trait locus (QTL) for resistance against *P. neglectus* was genetically mapped using a population of Excalibur/Kukri doubled haploid lines. This QTL coincides in position with the rust resistance gene(s) *Lr20/Sr15*, the phytoene synthase gene *Psy-A1* and 10 molecular markers, including five new markers designed using wheat-rice comparative genomics and wheat expressed sequence tags. Two of the new markers are suitable for use as molecular diagnostic tools to distinguish plants that carry *Rlnn1* and *Lr20*/*Sr15* from those that do not carry these resistance genes.

**Conclusions:**

The genomic location of *Rlnn1* was confirmed to be in the terminal region of the long arm of chromosome 7A. Molecular markers were developed that provide simple alternatives to costly phenotypic assessment of resistance against *P. neglectus* in wheat breeding. In Excalibur, genetic recombination seems to be completely suppressed in the *Rlnn1* region.

## Background

The root lesion nematode *Pratylenchus neglectus* infects a wide range of host plants, including wheat (*Triticum aestivum* L.) and crops that are grown in rotation with wheat. As a migratory endoparasite, *P. neglectus* moves in and out of roots, feeding as it moves through the root cortex. It causes lesions on roots, stunts plant growth and can significantly reduce crop yield. Plants that reduce the nematode population in root systems and in the soil are considered to be resistant. Resistant crop species and cultivars are valuable in crop rotations because they reduce the threat to subsequent crops.

The Australian wheat cultivars Excalibur and Krichauff have long been known to be at least moderately resistant against *P. neglectus*[1,2]. Williams et al. [3] attributed the *P. neglectus* resistance of these cultivars to a locus (*Rlnn1*) on the long arm of chromosome 7A (7AL). They estimated *Rlnn1* to be 9.1 cM distal to the gene *Lr20*, which confers resistance against leaf rust (*Puccinia triticina*). *Lr20* has in turn been reported to co-segregate with *Sr15* and *Pm1*[4,5], which confer resistance against stem rust (*Puccinia graminis* f. sp. *tritici*) and powdery mildew (*Blumeria graminis* f. sp. *tritici*), respectively.

Evaluation of resistance against *P. neglectus* can be laborious and costly, requiring replicated inoculated trials involving counting of nematodes (e.g., [3]) or the extraction of DNA from soil and root systems, followed by real-time polymerase chain reaction (PCR) to estimate the quantity of *P. neglectus* DNA [6]. Molecular markers for *Rlnn1* could therefore be very useful selection tools in wheat breeding, allowing phenotyping resources to be allocated only to progeny that had been pre-selected as likely to carry the resistance allele. Williams et al. [3] suggested conversion of the restriction fragment length polymorphism (RFLP) marker *cdo347* and the amplified fragment length polymorphism (AFLP) marker *AGC/CCT179*, both of which they had reported to be at the same position as *Lr20*, into PCR-based assays for use in marker-assisted selection of *Rlnn1*. To our knowledge, no such assays have been developed. This may have been because the reported distance of 9.1 cM seemed too large for such markers to be very useful in wheat breeding.

The phytoene synthase locus *Psy-A1* is also in the distal region of 7AL [7]. At this locus, there are multiple alleles, conferring different levels of yellow pigment in wheat grain and flour [8-10]. Krichauff and several other cultivars with moderate resistance against *P. neglectus* have been reported to carry either the *Psy-A1s* or *Psy-A1t* allele [8,9], both of which are associated with high levels of yellow pigment in flour. Wheat breeders in Australia are interested in developing cultivars with both white flour and resistance against *P. neglectus*, but, to our knowledge, they have not identified any materials with this combination of traits.

In the research reported here, a large mapping population was used to map *Rlnn1* relative to molecular markers and to the genes *Lr20*, *Sr15* and *Psy-A1*, and new markers were developed and validated for use in selection for resistance against *P. neglectus.*

## Results

### Resistance against leaf rust, stem rust and *Pratylenchus neglectus*

Evaluation of disease responses against the *P. triticina* pathotype 104–2,3,6,7 and the *P. graminis* f. sp. *tritici* pathotype 98–1,2,(3),5,6 indicated that Excalibur and 98 Excalibur/Kukri doubled haploid (DH) lines carry *Lr20/Sr15* resistance, while Kukri and 74 other Excalibur/Kukri DH lines are susceptible to both rust pathogens. No lines were observed to be resistant against one pathogen and susceptible to the other, indicating that if *Lr20* and *Sr15* are two genes (not a single pleiotropic gene), they are in complete coupling linkage in the Excalibur/Kukri population.

**Figure 1:**
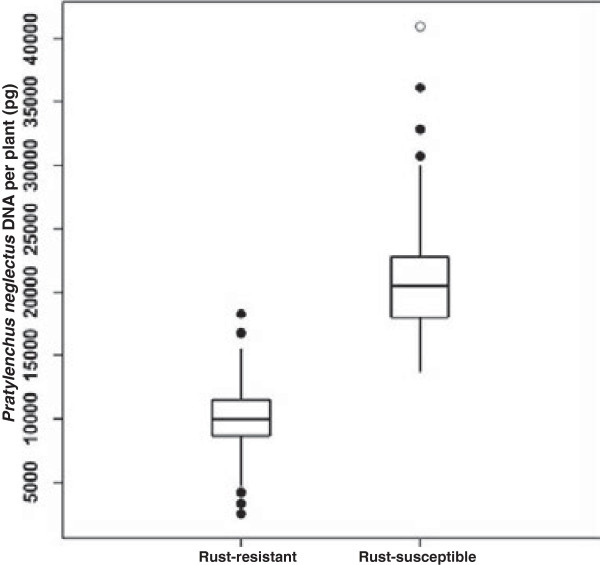
***Pratylenchus neglectus *resistance of rust-resistant and rust-susceptible Excalibur/Kukri doubled haploid lines.** Classification of lines as carrying or not carrying *Lr20/Sr15* rust resistance was based upon disease responses observed after inoculation of seedlings with appropriate pathotypes of *Puccinia triticina* and *Puccinia graminis* f. sp. *tritici.* Evaluation of resistance against the root lesion nematode *Pratylenchus neglectus* was based upon estimation of the quantity of *P. neglectus* DNA in the roots of inoculated wheat plants. The box spans the interquartile range of the estimated quantity of *P. neglectus* DNA per plant, the horizontal line within each box indicates the median. The whiskers extend to show the spread of data within the ‘inner fence’ (1.5 times the interquartile range beyond the quartiles). The solid black dots represent outliers within the ‘outer fence’ (3 times the interquartile range beyond the quartiles). The white dot represents an outlier outside of the ‘outer fence’.

The estimated quantity of *P. neglectus* DNA in the roots of inoculated plants, was 27,553 pg per plant for Kukri, but only 11,447 pg per plant for Excalibur. Quantitative variation for this measure of nematode resistance was observed within both the rust-resistant and rust-susceptible categories of Excalibur/Kukri DH lines (Figure 1), with the amount of *P. neglectus* DNA detected being significantly (*t* = 16.4, *p* < 0.001) lower for rust-resistant lines (9,959 pg) than for rust-susceptible lines (21,428 pg). Greater variability was observed for the more susceptible class than for the more resistant class (Figure 1), probably because of inoculation failures (‘escapes’) broadening the phenotypic range observed for susceptible lines. Failure of inoculation of individual plants (whether genetically resistant or susceptible) would be much more likely to occur than high multiplication of nematodes on genetically resistant plants.

### Genetic mapping

**Figure 2:**
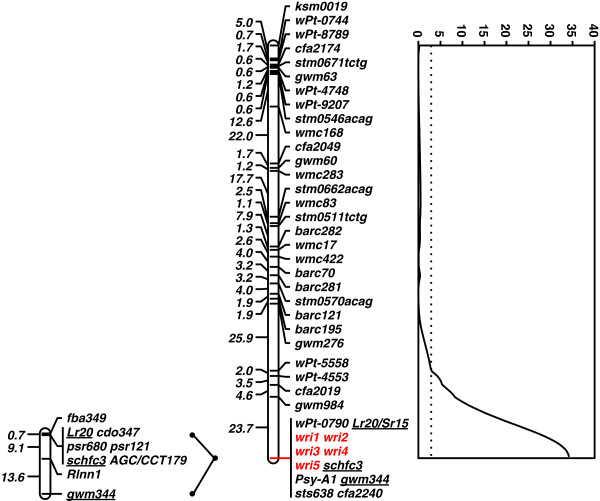
**Genetic map of chromosome 7A showing *Rlnn1, Lr20/Sr15, Psy-A1 *and molecular marker loci.** From left to right: a map showing the relative locations of the *Pratylenchus neglectus* resistance locus *Rlnn1*, the leaf rust resistance locus *Lr20* and seven molecular markers, as reported by Williams et al. [3]; a map showing the relative locations of the resistance locus *Lr20/Sr15*, the phytoene synthase gene *Psy-A1* and 39 molecular markers mapped on the chromosome 7A using a population of Excalibur/Kukri doubled haploid lines; and a graph showing a LOD test-statistic scan from simple interval mapping of resistance against the root lesion nematode *Pratylenchus neglectus* in the Excalibur/Kukri population, with resistance evaluated based upon estimation of the quantity of *P. neglectus* DNA in the roots of inoculated wheat plants. The names of three loci common to both maps are underlined. The names of five newly developed EST-based markers are shown in red.

Analysis of data from the Excalibur/Kukri mapping population, resulted in a high-quality genetic linkage map of chromosome 7A (Figure 2; Additional file 1), with *Lr20/Sr15* and *Psy-A1* collocating with each other and with the sequence tagged site (STS) markers *schfc3* and *sts638,* the simple sequence repeat (SSR) markers *gwm344* and *cfa2240* and the Diversity Arrays Technology (DArT™) marker *wPt-0790* at the distal end of 7AL. Sequencing of the products amplified with *PSY7A5_F/R* primers [9] indicated that Excalibur carries the *Psy-A1t* (‘very yellow’) allele (see Additional file 2), while amplification with *csPSY* primers [8] confirmed that Kukri carries the *Psy-A1p* (‘pale yellow’) allele.

With QTL analysis using the estimated quantity of *P. neglectus* DNA per plant as the trait value, *Rlnn1* was detected as a highly significant QTL (peak LOD = 34; Figure 2) at the same position as *Lr20*, *Sr15*, *Psy-A1* and the collocating STS, SSR and DArT markers. This locus accounted for 60% of the observed phenotypic variation.

### Development of new markers based on comparative analysis between wheat and rice

Since the physical locations of the SSR markers *cfa2240* (in the above-mentioned terminal marker cluster) and *cfa2019* (28.3 cM proximal to the cluster) had both previously been assigned to the wheat deletion bin 7AL16-0.86-0.90 [11], 42 unique expressed sequence tags (ESTs) from that deletion bin and 45 unique ESTs from the deletion bin 7AL18-0.90-1.00 were selected for use in comparative genomic analysis with rice (see Additional file 3). Of these 87 ESTs, 60 had BLASTN E-values less than e-10. Consistent with previous evidence of synteny between the distal part of 7AL and chromosome 6 of rice [12], more than half of these ESTs were similar to sequences in the terminal region of rice chromosome 6 (19 of 30 ESTs from 7AL16-0.86-0.90, and 15 of 30 ESTs from 7AL18-0.90-1.00).

Similarly, the probe sequences of three RFLP markers that had previously been reported to be linked with *Rlnn1, Lr20* and/or *Pm1*[3,4] showed high similarity with predicted genes in the terminal region of rice chromosome 6: PSR148 with LOC_Os06g51150 (1.2e-260), PSR680 with LOC_Os06g49380 (5.4e-103), and CDO347 with LOC_Os06g51270 (1.2e-98). The CDO347 sequence also had a significant BLASTN hit (5.7e-88) on rice chromosome 2 (LOC_Os02g58560). For PSR121, a probe that had been used to map another RFLP near *Rlnn1, Lr20* and *Pm1*[3,4] the most significant hit (7.6e-161) was on rice chromosome 5 (LOC_Os5g31140).

**Figure 3:**
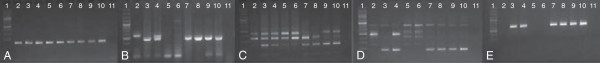
**Amplicons of markers *wri1*, *wri2, wri3, wri4 *and *wri5 *separated by agarose gel electrophoresis. (A)***wri1*, **(B)***wri2,***(C)***wri3,***(D)***wri4,***(E)***wri5,* (1) GeneRuler® 100 bp DNA ladder (Thermo Fisher Scientific Inc.), (2) Excalibur, (3) Kukri, (4) 1:1 mixture of Excalibur and Kukri DNA (artificial heterozygote), the Chinese Spring nullisomic-tetrasomic lines (5) CS N7A-T7B, (6) CS N7A-T7D, (7) CS N7B-T7A, (8) CS N7B-T7D, (9) CS N7D-T7A, (10) CS N7D-T7B and (11) water.

**Figure 4:**
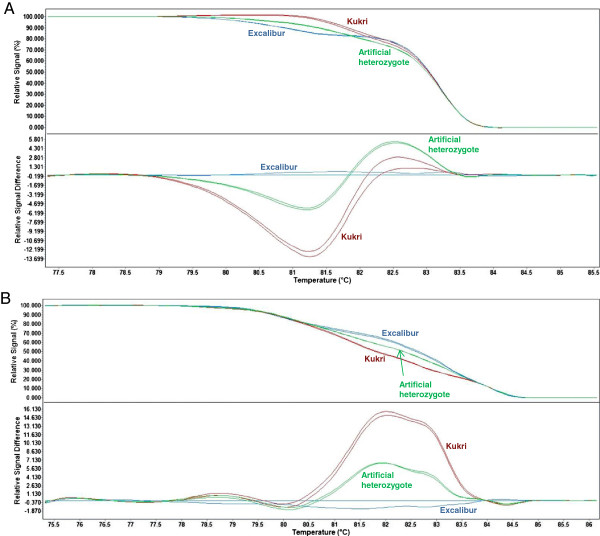
**High-resolution melting curves for amplicons of markers *wri1 *(A) and *wri3 *(B).** Above: Normalised and shifted melting curves; Below: Normalised and temperature-shifted difference plot. Curves are shown for products amplified from genomic DNA of Excalibur, Kukri and a 1:1 mixture of Excalibur and Kukri DNA (artificial heterozygote).

Given the evidence that the terminal region of rice chromosome 6 is syntenic with the *Rlnn1* region of wheat chromosome 7A, sequences from the terminal region of rice chromosome 6 were used to retrieve orthologous wheat ESTs and primers were designed to flank predicted introns. Analysis of PCR products amplified with primers designed from five wheat ESTs [GenBank:BE445506, GenBank:BE445653, GenBank:BF484041, GenBank:CN010180, GenBank:BF483039] detected readily assayable polymorphisms between the parents Excalibur and Kukri:

1. **BE445506:** The primer pair BE445506_F/R amplified products of approximately 150 bp from both parents and from an artificial heterozygote (a 1:1 mixture of Excalibur and Kukri DNA) (Figure 3A). Similar amplicons were obtained for all six Chinese Spring group-7 nullisomic-tetrasomic lines (CS N7A-T7B, CS N7A-T7D, CS N7B-T7A, CS N7B-T7D, CS N7D-T7A and CS N7D-T7B), indicating that this primer pair is not genome-specific. With high-resolution melting analysis, a polymorphism between Excalibur and Kukri was detected, providing a codominant marker assay (*wri1*) (Figure 4A).

2. **BE445653:** The primer pair BE445653_F/R (*wri2*) amplified 7A-specific products with differing sizes from Excalibur (586 bp) and Kukri (512 bp). This length polymorphism can be scored visually after electrophoretic separation on agarose gels (Figure 3B), providing a codominant marker assay. Sequencing of the amplicons revealed that the 74-bp length polymorphism between Excalibur and Kukri is due to the net effect of a number of insertion/deletion polymorphisms, which range in length from 1 to 30 nucleotides. Sequence polymorphisms between Excalibur and Kukri were also observed at 80 nucleotide sites (see Additional file 4).

3. **BF484041:** The primer pair BF484041_F/R (*wri3*) amplified three distinct products from Kukri (approximately 250 bp, 450 bp and 350 bp which are apparently specific to chromosomes 7A, 7B and 7D respectively). In Excalibur, only the 7B- and 7D-specific products showed strong amplification (Figure 3C). With high-resolution melting analysis, Excalibur, Kukri and an artificial heterozygote (a 1:1 mixture of Excalibur and Kukri DNA) each had a distinct melting curve, providing a codominant marker assay (Figure 4B).

4. **CN010180**: The primer pairs CN010180_F1/R and CN010180_F2/R amplified 7A-specific products from Excalibur and Kukri, respectively. The two clearly visible amplicons were of similar size (approximately 175 bp). With sequential loading of the two PCR products onto gels, the two complementary allele-specific primer pairs (CN010180_F1/R and CN010180_F2/R) provided a codominant marker assay (*wri4*) (Figure 3D).

5. **BF483039**: The primer pair BF483039A_F2/BF483039_cpR1, obtained from the Wheat SNP Database [13,14] amplified a product of approximately 600 bp from Kukri, but no product from Excalibur, providing a 7A-specific dominant marker (*wri5*, Figure 3E).

When assayed on the Excalibur/Kukri DH lines, all five EST-based markers co-segregated with *gwm344*, *cfa2240*, *wPt-0790*, *schfc3*, *sts638*, *Psy-A1*, *Lr20* and *Sr15*.

### Molecular marker genotypes of resistant and susceptible wheat cultivars

**Figure 5:**
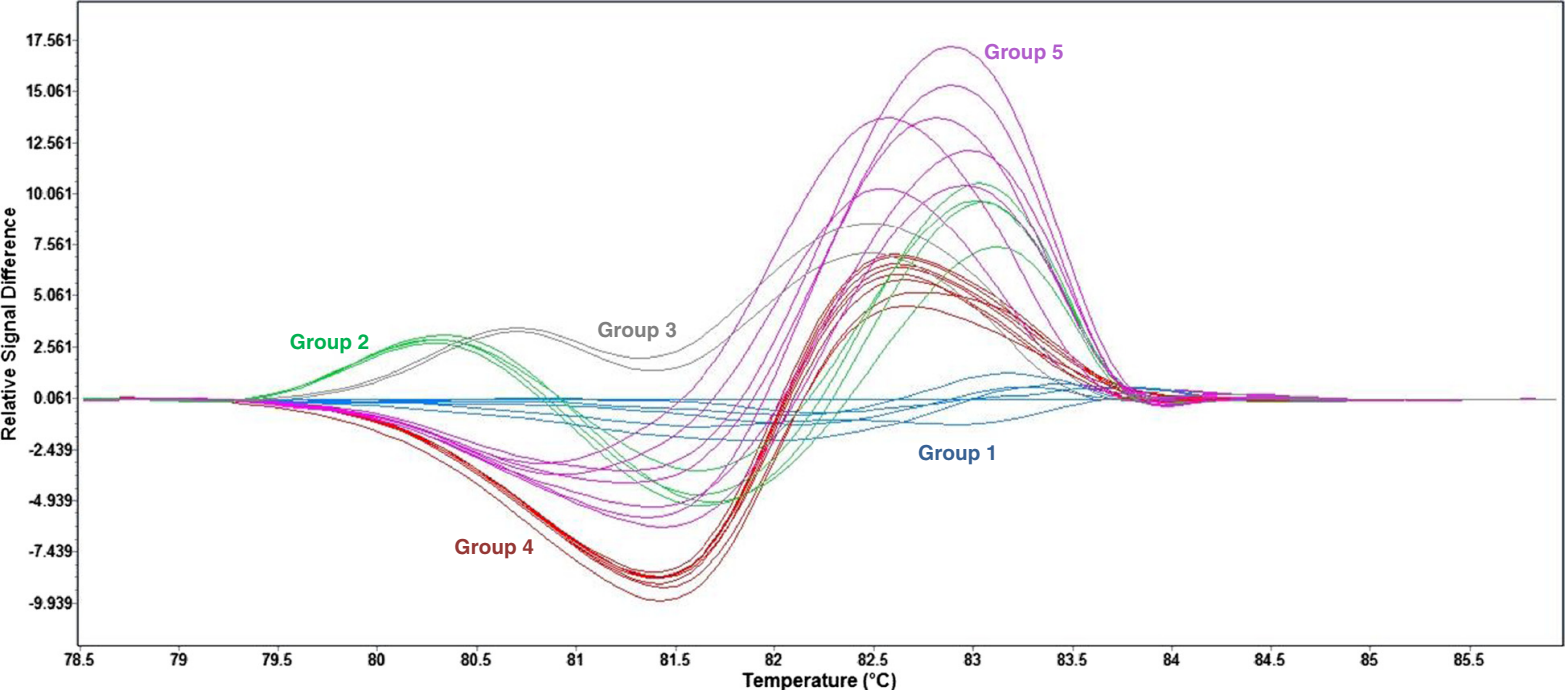
**High-resolution melting curves for amplicons of the co-dominant marker *wri1*.** Normalized and temperature- shifted difference plot for products amplified from genomic DNA of, Excalibur and 11 other cultivars known to carry *Rlnn1* and/or known to carry *Lr20*/*Sr15* (Group 1: Excalibur, Bindawarra, Bowie, Vectis, Wyalkatchem, Orion; Group 2: Aroona, Tatiara, Thew, Cascades; Group 3: BT- Schomburgk, Krichauff) and Kukri and 14 other cultivars known to lack *Rlnn1* and/or known to lack *Lr20*/*Sr15* (Group 4: Kukri, Sunbri, Calingiri, Sunlin, Sunvale, AGT Scythe, Annuello, Machete; Group 5: Sunstate, Chara, Babbler, Hartog, Buckley, Gladius, Yitpi).

When assayed on a panel of 25 wheat cultivars, the markers *wri2*, *wri3*, *wri4,* and *wri5* all clearly distinguished Excalibur and 11 other resistant cultivars (i.e., known to have moderate resistance against *P. neglectus* and/or to carry *Lr20*/*Sr15*) from Kukri and 14 other susceptible cultivars (i.e., known to be susceptible to *P. neglectus* and to lack *Lr20/Sr15* resistance). Marker *wri1* was able to distinguish Excalibur from Kukri, and it additionally detected polymorphisms within the resistant and susceptible classes, for which there were three distinct melting curves among the resistant cultivars and at least two others among the susceptible cultivars (Figure 5). This variation within phenotypic classes may reflect sequence polymorphism in homoeologous regions on chromosomes 7B and 7D.

With the *csPSY* marker, all 11 resistant cultivars provided the same result as Excalibur, indicating the presence of the *Psy-A1s* or *Psy-A1t* (‘very yellow’) alleles. None of the susceptible cultivars gave this result. With sequencing of the region in which the *Psy-A1s* or *Psy-A1t* alleles have been reported to differ, it was confirmed that all 11 resistant cultivars carry the *Psy-A1t* allele [GenBank:HM006895] (see Additional file 2). With *BstN1* restriction of the *csPSY* amplicon, each of the susceptible cultivars was classified as carrying either the *Psy-A1p* (‘pale yellow’) or *Psy-A1e* (‘white’) allele.

## Discussion

Data on 182 progeny lines derived from a cross between Excalibur and Kukri wheat were used to map the root lesion nematode resistance locus *Rlnn1* as a QTL on 7AL, at a position that coincides with several molecular markers and with the rust resistance loci *Lr20* and *Sr15*. As expected, the allele from Excalibur conferred greater resistance. For *P. neglectus* and other parasitic nematodes, resistance refers to the ability of the plant to limit nematode multiplication and thereby reduce the nematode population in the soil. In field experiments, measures of resistance require data on initial and final nematode populations. In inoculated experiments such as the one conducted here, estimates of the final populations are sufficient, given that the initial population is the same for all plants. In this experiment, *P. neglectus* DNA was quantified in the root systems only (and not in the surrounding soil). Accordingly, there could be some unknown confounding from differences in the size of the root system. Although root mass was not recorded in this experiment, no obvious differences in root size or density were noticed. Given that the moderate resistance of Excalibur was originally detected in field experiments [1,2] and that it has been repeatedly classified as moderately resistant using a range of methods, its resistance cannot be dismissed as simply an artefact of any variation in root size.

Our linkage map can be compared to a previously published map [3] based on the relative positions of the SSR marker *gwm344*, the rust resistance gene *Lr20* and the STS marker *schfc3* (Figure 2). In addition, the positions of the new markers *wri3* and *wri4* can be compared with the position of the RFLP locus *cdo347*. This is because *wri3* and *wri4* were designed based on EST sequences [GenBank:BF484041 and GenBank:CN010180] with high similarity (1.0e-56 and 4.0e-113, respectively) to the rice gene LOC_Os06g51270, which was the most significant hit for the CDO347 probe.

On the Excalibur/Kukri map, the *Rlnn1* QTL and four molecular markers (*gwm344*, *schfc3, wri3* and *wri4*) all collocate with *Lr20*. In the earlier map [3], *schfc3* and *cdo347* collocated with *Lr20,* but *gwm344* was 22.7 cM distal to *Lr20* and *Rlnn1* was about halfway between *Lr20* and g*wm344*. These discrepancies are probably due to differences in sample size and methodology. The original estimate of the position of *Rlnn1*[3] relied upon data from three small samples of progeny lines (25, 30 and 31 lines), each derived from a different cross. Each line was classified as either resistant or susceptible to *P. neglectus* and a consensus mapping approach was used to map *Rlnn1* as a discrete locus. With that approach and those sample sizes, any misclassification of lines or mis-scoring of markers could have considerably expanded the estimated distance between loci. In our work, trait data for *P. neglectus* resistance, which exhibited bimodal quantitative variation (Figure 1), were used to map *Rlnn1* as a QTL, without any attempt to classify the lines into distinct categories.

We detected a very strong association of resistance against *P. neglectus* with *Lr20/Sr15, Psy-A1* and 10 collocating molecular markers (*gwm344*, *cfa2240*, *schfc3*, *wPt-0790*, *sts638*, *wri1*, *wri2*, *wri3*, *wri4* and *wri5*). Of these, *sts638* had already been recommended [4] for selection of resistance conferred by *Lr20* and/or *Pm1*, but it has the disadvantage of being a dominant marker. The markers *schfc3*, *wPt-0790* and *wri5* are also dominant, as is *wri3* when assayed using gel electrophoresis with scoring of the presence or absence of a 250-bp chromosome 7A-specific amplicon. The markers *csPSY*, *gwm344*, *cfa2240*, *wri2* and *wri4* can all be assayed as codominant markers using electrophoresis, while *wri1* and *wri3* can be assayed as codominant markers using high-resolution melting analysis. We confirmed that *csPSY, wri2*, *wri3*, *wri4* and *wri5* all distinguish Excalibur and 11 wheat cultivars that are known to have moderate resistance against *P. neglectus* and/or to carry *Lr20*/*Sr15,* from Kukri and other 14 wheat cultivars that lack the resistance conferred by *Rlnn1* and *Lr20/Sr15*.

For marker-assisted selection of the root lesion nematode resistance locus *Rlnn1*, we recommend *wri2* for gel electrophoresis (Figure 3B) and *wri3* for high-resolution melting analysis (Figure 4B). The 7A-specific marker *wri2* is suitable for electrophoretic separation because it involves a substantial (74-bp) length polymorphism, whilst *wri3* can be scored as a high-throughput codominant marker using high-resolution melting analysis. Either of these markers would also be suitable for selection of the resistance genes *Lr20/Sr15* and *Pm1*. They both clearly distinguish between all of the resistant and susceptible materials that we have assayed, so they are likely to be useful across a wide range of wheat breeding germplasm.

Other loci within the wheat genome have also been reported to carry sets of co-segregating genes conferring resistance against rusts and powdery mildew. These include the *Lr34/Yr18/Pm38* locus on chromosome 7D, *Lr46/Yr29/Pm39* locus on chromosome 1B and *Lr67/Yr46* locus on chromosome 4D [15-21]. For the *Lr34/Yr18/Pm38* locus, a single gene encoding a protein resembling an adenosine triphosphate-binding cassette (ABC) transporter has been determined to confer resistance against leaf rust, stripe rust (caused by *Puccinia striiformis* f. sp. *tritici*) and powdery mildew [22]. For *Lr20/Sr15* and *Pm1,* McIntosh [23] found that changes to the *Lr20* disease reaction were always accompanied by changes to the *Sr15* reaction, while changes to *Pm1* were independent, indicating that the genetic determinant of *Pm1* resistance is not the same as for *Lr20/Sr15*. There is no particular reason to assume that the *Rlnn1* resistance effect is due to the same gene(s) as the *Lr20/Sr15* or *Pm1* resistance effects.

While coupling-phase linkage of alleles conferring resistance against multiple pathogens may be advantageous, the linkage of resistance alleles with a *Psy-A1* allele that confers high levels of yellow pigment in wheat flour is disadvantageous for markets that favour white flour. Recombinant progeny carrying the nematode resistance gene in combination with a low-pigment allele of *Psy-A1* would be useful in wheat breeding. We have not found any such recombinants. Neu et al. [4] have suggested that *Lr20* and *Pm1* are in a region of suppressed recombination. They proposed that this could be due to an alien introgression or a genetic rearrangement. Based on the results presented here, it seems likely that *Rlnn1* and *Psy-A1* are also within the region of suppressed recombination. Based on previously reported sequence similarity of the *Psy-A1t* allele with B-genome alleles [9], it seems possible that chromosome 7A of Excalibur and the other resistant cultivars carries an ancestral translocation from the terminal region of the long arm of chromosome 7B.

Both the observed collocation of the *Rlnn1* QTL with *Psy-A1* (Figure 2) and the hypothesis that these loci are in a region of suppressed recombination are consistent with the experience of wheat breeders in Australia, who have not (to our knowledge) been able to combine *Rlnn1* resistance with white flour colour. In addition to limiting the opportunity to obtain favourable recombinants between *Rlnn1* and *Psy-A1* for wheat breeding purposes, suppression of recombination could impede positional cloning of *Rlnn1*, *Lr20/Sr15* and *Pm1*.

## Conclusions

The research reported here has clarified the position of the *Rlnn1* locus for resistance against the root lesion nematode *P. neglectus*, by using a large wheat population to map *Rlnn1* as a QTL, rather than relying upon consensus mapping from small samples. The approach adopted here has clearly shown that *Rlnn1* is very closely linked with the rust resistance gene(s) *Lr20* and *Sr15*, the *t* allele of the phytoene synthase gene *Psy-A1* and several molecular markers. Some of the molecular markers developed here are useful for wheat breeding; they provide simple alternatives to costly phenotypic assessment of resistance against *P. neglectus* and they have been confirmed to be diagnostic across a panel of wheat cultivars. The results reported here are consistent with the hypothesis that materials with *Rlnn1* resistance carry a chromosome rearrangement on 7AL and that this suppresses genetic recombination in this region.

## Methods

### Plant materials

The plant materials used here included the Australian wheat cultivars Excalibur (RAC177(*Sr26*)/Uniculm492//RAC311S; released by the University of Adelaide in 1991) and Kukri (76ECN44/76ECN36//RAC549; Madden/6*RAC177; released by the University of Adelaide in 1999), 182 DH lines produced from the F_1_ generation of a cross between Excalibur and Kukri, a panel of 25 other wheat cultivars (sourced from the Australian Winter Cereals Collection) and six Chinese Spring nullisomic-tetrasomic lines (CS N7A-T7B, CS N7A-T7D, CS N7B-T7A, CS N7B-T7D, CS N7D-T7A and CS N7D-T7B, developed by Sears [24]).

The 182 Excalibur/Kukri DH lines were selected from 233 such lines for which molecular markers had previously been assayed [25]. The remaining 51 DH lines were excluded based on examination of the molecular marker data: 43 because they seemed to be genetically identical to other lines in the population, 6 because the DNA samples representing those lines exhibited more than one allele for numerous markers, indicating possible contamination of the lines or DNA samples, and 2 due to possible errors in DNA extraction.

Among the 25 wheat cultivars in the panel, 11 (Aroona, Bindawarra, Bowie, BT-Schomburgk, Wyalkatchem, Cascades, Krichauff, Thew, Tatiara, Orion and Vectis) are known to have moderate resistance against *P. neglectus* and/or to carry *Lr20*/*Sr15*, while the other 14 (AGT Scythe, Annuello, Babbler, Buckley, Calingiri, Chara, Gladius, Hartog, Machete, Sunbri, Sunlin, Sunstate, Sunvale and Yitpi) are susceptible to *P. neglectus* and do not carry *Lr20/Sr15.*

### Evaluation of resistance against leaf rust, stem rust and *Pratylenchus neglectus*

Resistance against rust pathogens was evaluated using the methods described by Bariana and McIntosh [26]. Two sets of seedlings were sown, one for inoculation with the *P. triticina* (Pt) pathotype 104–2,3,6,7 (PBI culture no. 231) and one for inoculation with the *P. graminis* f. sp. *tritici* (Pgt) pathotype 98–1,2,(3),5,6 (PBI culture no. 279). For each pathogen, 176 Excalibur/Kukri DH lines (six of the lines that had been genotyped were excluded due to insufficient seed availability or poor germination) were sown in 9-cm plastic pots (five seeds per line and four lines per pot) filled with a mixture of pine bark and river sand. Sown pots were fertilized with balanced fertilizer Aquasol® (10 g/10 L for 100 pots). Seedlings were raised in a controlled-environment microclimate room maintained at 20 ± 2°C. At 7 d after sowing, urea was applied at the same rate as the Aquasol®. At the two-leaf stage (10 to 12 d after sowing), urediniospores suspended in light Shellsol T® mineral oil were atomised on the seedlings using a hydrocarbon pressure pack. The Pt-inoculated seedlings were incubated for 24 h in a misting room fitted with an ultrasonic humidifier. The Pgt-inoculated seedlings were incubated for 48 h under natural light conditions at 18 ± 2°C on water-filled steel trays covered with plastic hoods. Seedlings were then transferred to a microclimate room maintained at 18 ± 2°C. Infection type assessments of each line were made 14 d after inoculation using the 0–4 scales described by McIntosh et al. [27]. Each line was classified as resistant or susceptible to leaf and stem rust.

Evaluation of resistance against *P. neglectus* was carried out by the South Australian Research and Development Institute Root Disease Testing Service, Adelaide [6]. Plants were grown in a partially replicated experiment using four blocks sown at weekly intervals. Thirteen lines had to be excluded from this experiment due to insufficient availability of viable seed. Excalibur, Kukri and each of 170 Excalibur/Kukri DH lines, were each assigned to one block, while each of 50 Excalibur/Kukri DH lines was assigned to two blocks. Seeds of Excalibur, Kukri and the Excalibur/Kukri DH lines were pre-germinated over a period of 2 d in a misting chamber maintained between 22 and 24°C. For each experimental unit, five pre-germinated seeds were sown in tubes (55 × 120 mm) containing 350 g of steam-pasteurised sand. These tubes were arranged in crates according to a pre-determined design, with each experimental unit consisting of a row of five adjacent tubes. Watering was carried out by flooding the tubes up to a 100-mm depth for 4 min, every 3 d.

The *P. neglectus* inoculum used in the study was sourced from plants grown in Cambrai soil in South Australia. The culture was maintained in carrot calli using methods modified from Moody et al. [28] and was subcultured every 90 d. The nematodes were extracted from the carrot calli using a mist chamber as described by Hooper [29]. Nematodes were counted in three 250-μL samples of extract and the inoculum was adjusted to a final concentration of 1,500 nematodes/mL by diluting with sterile water. One week after sowing, each tube was inoculated by making two 50-mm-deep holes close to the seedling and dividing a 1-mL aliquot of the inoculum between the two holes. After inoculation, watering was suspended for 3 d. On the second day after inoculation, 1.4 g of a slow-release fertiliser (Osmocote, Scotts Miracle-Gro, USA) was added to each tube and the sand surface was covered with a layer of plastic beads to reduce evaporation and mould development.

Eight weeks after inoculation, the plants were removed from the tubes. The roots were cut, washed with running water to remove all soil and dried at 60°C. For each plant, total DNA was extracted from air dried roots as has been described for soil DNA extraction [30,31]. Quantitative real-time PCR (qPCR) was performed on an ABI PRISM® 7900HT instrument using TaqMan® Minor Groove Binder probe (Applied Biosystems, Foster City, CA, USA). The TaqMan probe (6FAM- 5′- CATTGGGCTCAGAAAC −3′) was used at 200 nM final concentration with a forward primer (5′- CACGGACCAAGGAGTTTATCG −3′) and a reverse primer (5′-CGAGGACATGTTTCACTTTCATTG-3′), each at 400 nM final concentration. The qPCR reactions were prepared using the QuantiTect Probe PCR Master Mix (QIAGEN GmbH, Hilden, Germany). The PCR conditions were 50°C for 2 min; 95°C for 15 min followed by 40 cycles of 95°C for 15 s and 60°C for 1 min. The quantity of *P. neglectus* DNA per plant was calculated using a standard calibration curve obtained from 10-fold serial dilution series of purified *P. neglectus* genomic DNA.

The resulting phenotypic data were analysed using the REML directive in GenStat v15.3.0.9425 (VSN International Ltd., UK), employing a model that included random effects for the experimental block and the parental or DH line. Comparisons of the *P. neglectus* resistance between the rust-resistant and rust-susceptible groups were carried out by applying a two-sample t test to line best linear unbiased predictions (BLUPs) for *P. neglectus* DNA per plant, assuming unequal group variances.

### Wheat-rice comparative analysis and development of new markers from wheat ESTs

Because the physical locations of the *Rlnn1*-linked SSR markers *cfa2019* and *cfa2240* had previously been assigned to wheat deletion bin 7AL16-0.86-0.90 [11], 87 unique ESTs from the two terminal deletion bins 7AL16-0.86-0.90 and 7AL18-0.90-1.00 of wheat physical EST maps in GrainGenes database [32] were used as BLASTN queries to search the rice genome sequence at Rice Genome Annotation Project database [33,34]. In addition, probe sequences of the *Rlnn1* and/or *Lr20*/*Sr15*/*Pm1-*linked RFLP markers *cdo347*, *psr121*, *psr148,* and *psr680* from the GrainGenes database [32] were used as BLASTN queries against the GenBank *Triticum* EST database at NCBI [35]. BLAST hits with E-values less than e-40, were retrieved and used to query rice coding sequences at the Rice Genome Annotation Project database [33,34]. Once an orthologous region of the rice genome was identified, the sequences of predicted rice genes in that region were used as BLASTN queries to search the GenBank *Triticum* EST database at NCBI [35] for wheat orthologues. Sequence comparisons of the rice genes with their putative wheat EST orthologues were used to predict the positions of wheat introns, and PCR primers were designed to span predicted introns. In addition, primer sequences that had previously been designed for wheat ESTs assigned to 7AL16-0.86-0.90 and 7AL18-0.90-1.00 were obtained from the Wheat SNP Database [13,14]. Newly designed PCR primer pairs and those obtained from the SNP database (Table 1) were used to amplify products from genomic DNA of Excalibur and Kukri using PCR conditions described in Additional file 5. For each primer pair, the amplicons from Excalibur and Kukri were examined for polymorphism using electrophoresis on agarose gels, high-resolution melting analysis in a Roche LightCycler® 480 and/or sequencing.

**Table 1:** PCR markers designed based on five wheat expressed sequence tags (ESTs)

**Marker**	**EST**	**Forward primer (5′-3′)**		**Reverse primer (5′-3′)**	
*wri1*	BE445506	BE445506_F	TCAGACAATTTAGTGGATGCTCG	BE445506_R	CCGCAAAGTAGCACGCCTT
*wri2*	BE445653	BE445653_F	TGGACACTGGGTTCATCGAGGA	BE445653_R	AGCAAGCACACTAGCCACTCTGTTT
*wri3*	BF484041	BF484041_F	TTCTCGCTGCAAAACTATGGC	BF484041_R	TTCACAGATCCAGTGCTTATGTCG
*wri4*	CN010180	CN010180_F1	ATGGGGCCTCTTCGG	CN010180_R	GSAAACATCTGTCTGATTACARAATAAGAA
		CN010180_F2	ATGGGGCCTCTTCGC		
*wri5*	BF483039	BF483039A_F2	GCAGCTACTTTGAACCCTTCAG	BF483039_cpR1	CGACCGTCTTCCCGAACAGC

### Partial sequencing of *Psy-A1* and BE445653

The primer pair *PSY7A5_F/R*[9] was used to amplify a region of the gene *Psy-A1* from the cultivars Excalibur, Aroona, Bindawarra, Bowie, BT-Schomburgk, Wyalkatchem, Cascades, Krichauff, Thew, Tatiara, Orion and Vectis using conditions given in Additional file 5. PCR products were cleaned up using NucleoSpin® Gel and PCR clean-up kit (MACHEREY-NAGEL GmbH & Co. KG). Sequencing was carried out at Australian Genome Research Facility Ltd., Adelaide by capillary separation on the Applied Biosystems 3730xl DNA analyzer (Applied Biosystems, CA, USA). The sequences of Excalibur and 11 other cultivars were aligned to *Psy-A1t* allele sequence of the breeding line WAWHT2074 [GenBank:HM006895] [9] and *Psy-A1s* allele sequence of the cultivar Schomburgk [GenBank:EU649795] [8] with the multiple align module implemented in Geneious® 6.0.4 (Biomatters Ltd., New Zealand) with default settings.

Primer pair *wri2_F/R* was used to amplify a region of BE445653 from the cultivars Excalibur, Bowie, Wyalkatchem, Cascades, Krichauff, Kukri, Machete, AGT Scythe, Chara, Gladius, Buckley and Yitpi using the conditions described in Additional file 5. PCR purification, sequencing and sequence analysis and alignment were performed as described above.

### Genotyping of molecular markers

The STS markers *schfc3* (designed based on the STS marker *FC7*[36]) and *sts638*[4], the *Psy1-A1* marker *csPSY*[8] and five new markers (*wri1*, *wri2*, *wri3*, *wri4* and *wri5*, Table 1) were assayed on Excalibur, Kukri and each of the 182 Excalibur/Kukri DH lines, using PCR conditions described in Additional file 5. For markers *wri2, wri4, wri5, schfc3* and *csPSY,* genotypes were assayed on 2.0 to 2.5% agarose gels (see Additional file 5). For markers *wri1*, *wri3* and *sts638,* genotypes were assayed using high-resolution melting technology [37] in a Roche LightCycler® 480 using conditions reported in Additional file 5. Melting curves were analysed using the gene scanning module of Roche LightCycler® software version 1.5.0. The five new markers (*wri1, wri2, wri3, wri4* and *wri5*) and *csPSY* were also assayed on the panel of 25 wheat cultivars, on the Chinese Spring nullisomic-tetrasomic lines (CS N7A-T7B, CS N7A-T7D, CS N7B-T7A, CS N7B-T7D, CS N7D-T7A and CS N7D-T7B) and on an artificial heterozygote (created by combining equal amounts of Excalibur and Kukri 50-ng/μl DNA).

### Genetic mapping

For each rust pathogen, each Excalibur/Kukri DH line for which all plants were resistant (infection type of 0–2) was classified as carrying the Excalibur allele of the corresponding rust resistance gene and each line for which all plants were susceptible (infection type of 3–4) were classified as carrying the Kukri allele. Four lines that exhibited mixed responses (some resistant plants and some susceptible) were treated as having missing data for the rust resistance loci. Similarly, for each molecular marker, each DH line was classified as carrying the same allele as Excalibur or the same allele as Kukri. Six markers that had originally been mapped on chromosome 7A [25] were excluded due to ambiguous genotypic scores (*gdm125*, *gwm681*, *wPt-7299*, *wPt-2260* and *wPt-1259*) or a high frequency of missing data (*wPt-6668*). The newly assayed markers (*wri1*, *wri2*, *wri3*, *wri4*, *wri5*, *sts638* and *schfc3*), the phytoene synthase gene *Psy-A1* and the rust resistance locus *Lr20/Sr15* were added to the existing chromosome 7A genetic linkage map [25] using the ‘distribute’ function of MapManager QTXb20 [38] (search linkage criterion set at a LOD threshold of *p =* 1e-6; map distance estimation done using Kosambi mapping function [39]). The marker order was refined using RECORD [40] and the goodness-of-fit of the marker order was examined by plotting pairwise recombination fractions against LOD linkage statistics. Line BLUPs of the quantity of *P. neglectus* DNA per plant were used to map *Rlnn1* as a QTL on the resulting genetic map by interval mapping as implemented in Qgene 4.3.10 [41] with a step size of 1 cM and a significance threshold for detection of QTL set by performing 1000 permutations with a genome wide Type I error rate of 0.05. The final linkage map and QTL graph was plotted using MapChart v2.2 [42].

## Abbreviations

QTL: Quantitative trait locus; 7AL: Long arm of chromosome 7A; PCR: Polymerase chain reaction; RFLP: Restriction fragment length polymorphism; AFLP: Amplified fragment length polymorphism; STS: Sequence-tagged site; DH: Doubled haploid; SSR: Simple sequence repeat; DArT™: Diversity arrays technology; LOD: Logarithmic of odds; EST: expressed sequence tag; Pt: *Puccinia triticina*; Pgt: *Puccinia graminis* f. sp. *tritici*; qPCR: Quantitative real-time polymerase chain reaction; BLUP: Best linear unbiased prediction.

## Competing interests

The authors declare that they have no competing interests.

## Authors’ contributions

AM designed the methods used to quantify *P. neglectus* DNA in plant root systems. HK designed the experiment to evaluate the Excalibur/Kukri population for resistance against *P. neglectus*. JE analysed the phenotypic data from that experiment and constructed an initial genetic linkage map. HB evaluated the population for resistance against leaf rust and stem rust. ET conducted wheat-rice comparative analysis, designed new markers and assayed them on the population. DJ critically examined all genotypic and phenotypic data, assayed additional markers, reconstructed the linkage map, conducted QTL analysis, assayed selected markers on a panel of cultivars and interpreted the results. DJ and DM wrote the manuscript, with input from ET. KC supervised the marker design, genotyping and linkage mapping and DM provided overall supervision of the research. All authors read and approved the final manuscript.

## Authors’ information

DVJ and EJT are joint first authors.

## Supplementary Material

Additional file 1 Pairwise recombination fractions and LOD linkage plot of chromosome 7A. Click here for file

Additional file 2 **Alignment of sequences of *PSY7A5_F/R *** **amplicons from Excalibur and 11 other cultivars to the *Psy-A1t*** **allele sequence of the breeding line WAWHT2074 [GenBank:HM006895] and the** ***Psy-A1s*** **allele sequence of the cultivar Schomburgk [GenBank:EU649795].** Click here for file

Additional file 3 Rice BLASTN results using query sequences for ESTs from wheat deletion bins 7AL16-0.86-0.90 and 7AL18-0.90-1.00. Click here for file

Additional file 4 **Alignment of sequences of *wri2_F/R *** **amplicons from Excalibur, Kukri and 10 other cultivars.** Click here for file

Additional file 5 Primer sequences and conditions used for PCR and for separation of amplicons by gel electrophoresis and by high-resolution melting analysis. Click here for file
