# Supplementary figures and images for: Genetic mapping and marker development for resistance of wheat against the root lesion nematode Pratylenchus neglectus
Source: BMC Plant Biol. 2013 Dec 31;13:230. doi: 10.1186/1471-2229-13-230 (PMC3923441; doi:10.1186/1471-2229-13-230)

Additional file 1: Pairwise recombination fractions and LOD linkage plot of chromosome 7A

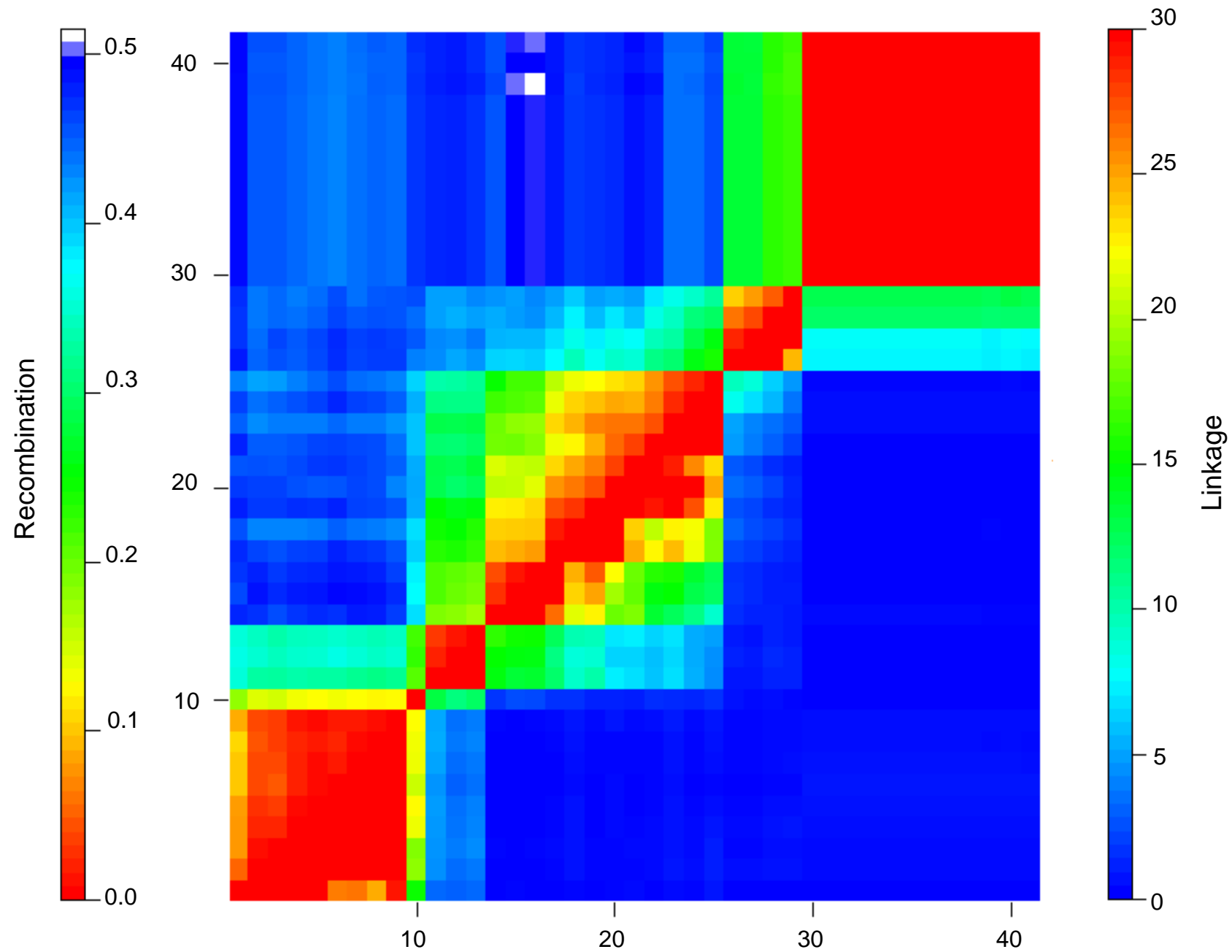

Supplement: Additional file 1 — Pairwise recombination fractions and LOD linkage plot of chromosome 7A. [file 1471-2229-13-230-S1.pdf]
