# Supplementary material for: Genetic mapping and marker development for resistance of wheat against the root lesion nematode Pratylenchus neglectus
Source: BMC Plant Biol. 2013 Dec 31;13:230. doi: 10.1186/1471-2229-13-230 (PMC3923441; doi:10.1186/1471-2229-13-230)

Additional file 4: Alignment of sequences of *wri2_F/R* amplicons from Excalibur, Kukri and 10 other cultivars

1. Full sequences of *wri2_F/R* amplicons from Excalibur and Kukri

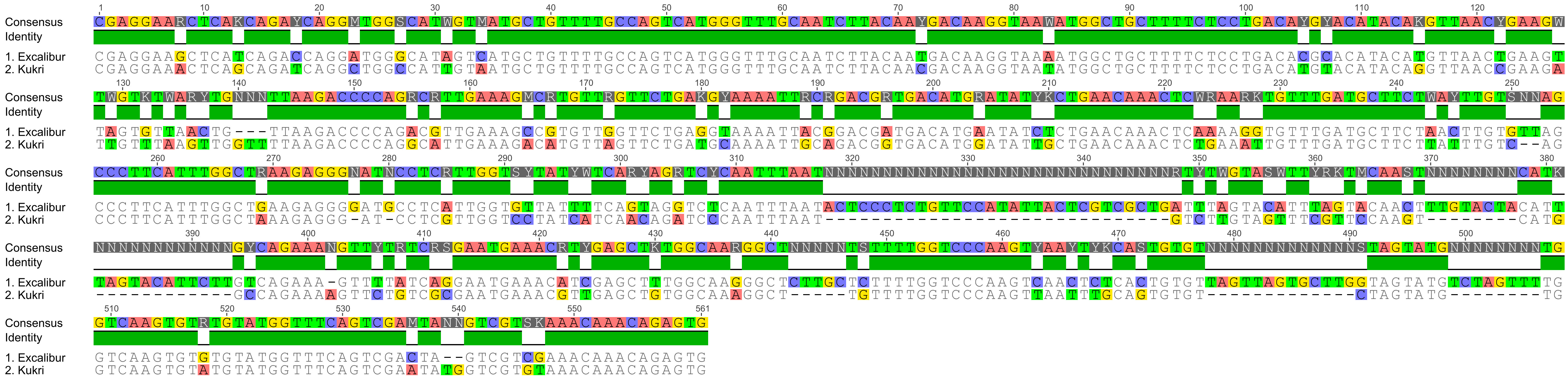

2. Partial sequences of *wri2_F/R* amplicons from Excalibur, Kukri and 10 other cultivars

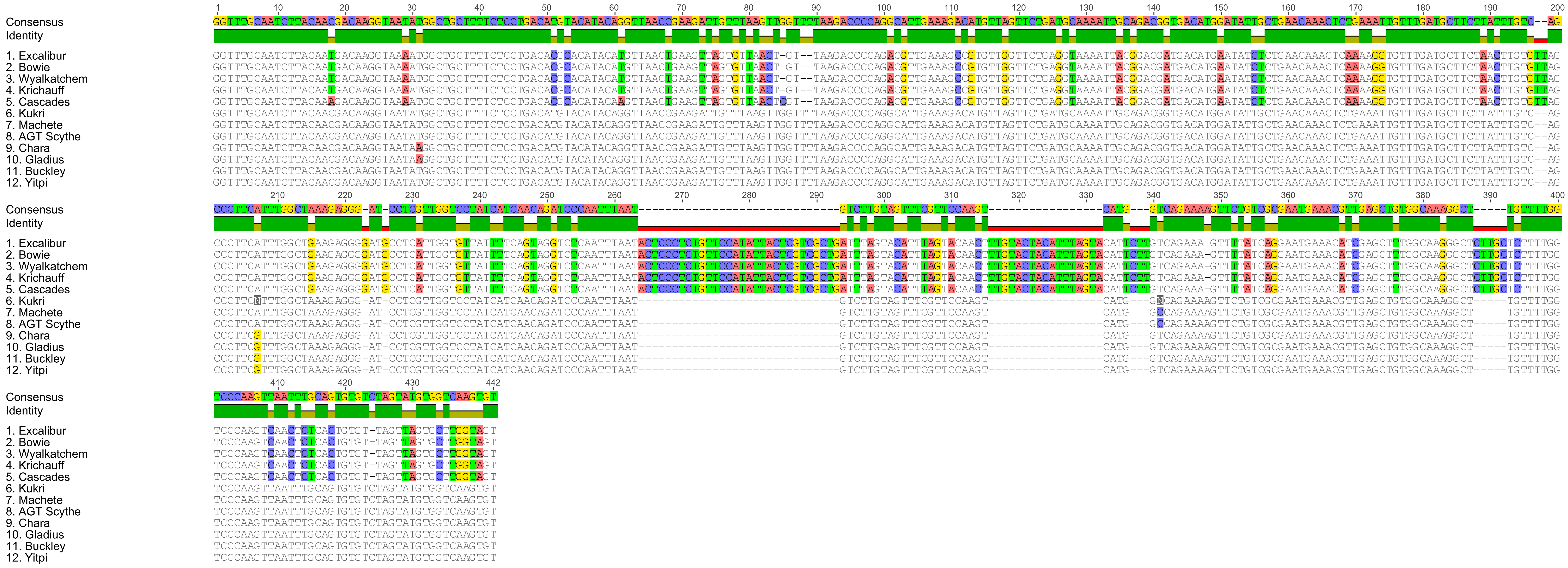

Supplement: Additional file 4 — Alignment of sequences of wri2_F/R amplicons from Excalibur, Kukri and 10 other cultivars. [file 1471-2229-13-230-S4.pdf]
