# Supplementary material for: Genetic mapping and marker development for resistance of wheat against the root lesion nematode Pratylenchus neglectus
Source: BMC Plant Biol. 2013 Dec 31;13:230. doi: 10.1186/1471-2229-13-230 (PMC3923441; doi:10.1186/1471-2229-13-230)
Supplement: Additional file 5 — Primer sequences and conditions used for PCR and for separation of amplicons by gel electrophoresis and by high-resolution melting analysis. [file 1471-2229-13-230-S5.pdf]

Additional file 5: Primer sequences and conditions used for PCR and for separation of amplicons by gel electrophoresis and by high-resolution melting analysis

1. Primer sequences

Primer name	Forward primer sequence (5` - 3`)	Reverse primer sequence (5` - 3`)	Reference
schfc3	TGCAGACCACCTCGGCTG	TAACAGCGGATATGATGG	Personal communication (Meredith Carter)
sts638	GCGGTGACTACACAGCGATGAAGCAATGAAA	GCGGTGACTAGTCCAGTTGGTTGATGGAAT	Neu et al. (2002)
csPSY	GGCCTTCTAAGTTGACCAG	GTCATGTCTGTTCTTCAGAGG	Howitt et al. (2009)
	GCCAAGCCGGTGTTCCGG	CCTCCTGCACCATACTGAAC	
PSY7A5	GCGGAGTGGTGACTGGTG	GGCGGTCTGAACTCTGAAGTG	Crawford et al. (2011)

2. Methods for agarose gel electrophoresis based assays

PCR reaction mixtures

PCR reaction mixture 1: *sts638*, *wri4*

	Final concentration
Template DNA	15 ng/µl
Immolase	0.015 U/µl
Immolase buffer	1 x
dNTPs	0.2 mM
$MgCl_2$	1.5 mM
Forward primer	0.5 µM
Reverse primer	0.5 µM

PCR reaction mixture 2: *schfc3*, *wri1*, *wri3*

	Final concentration
Template DNA	15 ng/µl
Immolase	0.015 U/µl
Immolase buffer	1 x
dNTPs	0.2 mM
$MgCl_2$	2.5 mM
Forward primer	0.5 µM
Reverse primer	0.5 µM

PCR reaction mixture 3: *wri2*

	Final concentration
Template DNA	20 ng/µl
Immolase	0.015 U/µl
Immolase buffer	1 x
dNTPs	0.2 mM
$MgCl_2$	2.5 mM
Bovine serum albumin (BSA)	0.1 mg/mL
Forward primer	0.5 µM
Reverse primer	0.5 µM

PCR reaction mixture 4: *wri5*

	Final concentration
Template DNA	15 ng/µl
Immolase	0.0375 U/µl
Immolase buffer	1 x
dNTPs	0.2 mM
$MgCl_2$	1.0 mM

Bovine serum albumin (BSA)	0.1 mg/mL
Forward primer	0.3 µM
Reverse primer	0.3 µM

PCR reaction mixture 5: *csPSY*

	Final concentration
Template DNA	15 ng/µl
Immolase	0.015 U/µl
Immolase buffer	1 x
dNTPs	0.2 mM
$MgCl_2$	2.5 mM
Forward primer	0.5 µM
Reverse primer	0.5 µM

Digestion of 5 µl of *csPSY* PCR product with *BstN1* (final reaction volume 10 µl)

	Final concentration
NEBuffer 2	1 x
BstN1	0.5 U/µl
Bovine serum albumin (BSA)	1 mg/mL

PCR programs

PCR program 1: *schfc3 (Tm-55), sts638 (Tm-62), wri1(Tm-60), wri3(Tm-60)* and *wri5(Tm-60)*

Step 1 - 95 °C for 10 min

Step 2 - 94 °C for 30 s

Step 3 – *Tm* °C for 30s

Step 4 - 72 °C for 1 min

Step 5 - Step 2 for 34 cycles

Step 6 - 72 °C for 5 min

Step 7 - End

PCR program 2: *wri2*

Step 1 - 95 °C for 10 min

Step 2 - 94 °C for 30 s

Step 3 – 60 °C (-0.5 °C/cycle) for 30s

Step 4 - 72 °C for 1 min

Step 5 – step 2 for 19 cycles

Step 6 - 94 °C for 30 s

Step 7- 50 °C for 30s

Step 8 - 72 °C for 1 min

Step 9 – Step 6 for 24 cycles

Step 10 - 72 °C for 5 min

Step 11 – End

PCR program 3: *csPSY*

Step 1 - 95 °C for 10 min

Step 2 - 94 °C for 30 s

Step 3 - 65 °C (-0.5 °C/cycle) for 30s

Step 4 - 72 °C for 45 s

Step 5 - step 2 for 19 cycles

Step 6 - 94 °C for 30 s

Step 7- 55 °C for 30 s

Step 8 - 72 °C for 45 s

Step 9 - Step 6 for 24 cycles

Step 10 - 72 °C for 5 min

Step 11 – End

Digestion with *BstN1*

60 °C for 2 hours

PCR program 4: *wri4*

Step 1 - 95 °C for 10 min

Step 2 - 94 °C for 30 s

Step 3 – 58 °C for 30s

Step 4 - 72 °C for 1 min

Step 5 - step 2 for 14 cycles

Step 6 - 94 °C for 10 s

Step 7 - 45 °C for 30s

Step 8 - Step 6 for 4 cycles

Step 9 - 94 °C for 10 s

Step 10 - 53 °C for 30s

Step 11 - 72 °C for 5 s

Step 12 - step 9 for 14 cycles

Step 13 - End

Gel electrophoresis conditions

schfc3, *sts638*, *wri1*, *wri4* and *wri5* – 2% agarose gel; 90V; 40 min

csPSY, *wri2* and *wri3* – 2.5% agarose gel; 90V; 60 min

3. Methods for high-resolution melting based assays

PCR mixtures

PCR reaction mixture 5: *sts638*

	Final concentration
Template DNA	15 ng/µl
Immolase	0.015 U/µl
Immolase buffer	1 x
dNTPs	0.2 mM
$MgCl_2$	1.5 mM
Forward primer	0.5 µM
Reverse primer	0.5 µM
Syto9 dye	2.0 µM

PCR reaction mixture 6: *wri1*, *wri3*

	Final concentration
Template DNA	15 ng/µl
Immolase	0.015 U/µl
Immolase buffer	1 x
dNTPs	0.2 mM
$MgCl_2$	2.5 mM
Forward primer	0.5 µM
Reverse primer	0.5 µM
Syto9 dye	2.0 µM

PCR program

PCR program 1 (see above): *sts638, wri1*, *wri3*

HRM conditions

Step 1 - 95°C for 30 s

Step 2 - (*Tm* - 2) °C for 30 s; ramp to 95 °C (+ 0.02 °C/s; 25 acquisitions/ °C)

Step 3 – 37°C for 1 s

4. Methods for partial sequencing of *Psy-A1*

PCR mixture

PCR reaction mixture 7: *PSY7A5*

	Final concentration
Template DNA	15 ng/µl
Immolase	0.015 U/µl
Immolase buffer	1 x
dNTPs	0.2 mM
$MgCl_2$	1.5 mM
Forward primer	0.4 µM
Reverse primer	0.4 µM
DMSO	6%

PCR program

PCR program 5: *PSY7A5*

Step 1 - 95 °C for 10 min

Step 2 - 95 °C for 15 s

Step 3 – 60 °C for 15 s

Step 4 - 72 °C for 30 s

Step 5 - Step 2 for 34 cycles

Step 6 - 72 °C for 7 min

Step 7 - End
