# Supplementary material for: Genetic mapping and marker development for resistance of wheat against the root lesion nematode Pratylenchus neglectus
Source: BMC Plant Biol. 2013 Dec 31;13:230. doi: 10.1186/1471-2229-13-230 (PMC3923441; doi:10.1186/1471-2229-13-230)

Additional file 2: Alignment of sequences of *PSY7A5_F/R* amplicons from Excalibur and 11 other cultivars to the *Psy-A1t* allele sequence of the breeding line WAWHT2074 [GenBank:HM006895] and the *Psy-A1s* allele seqeunce of the cultivar Schomburgk [GenBank:EU649795]

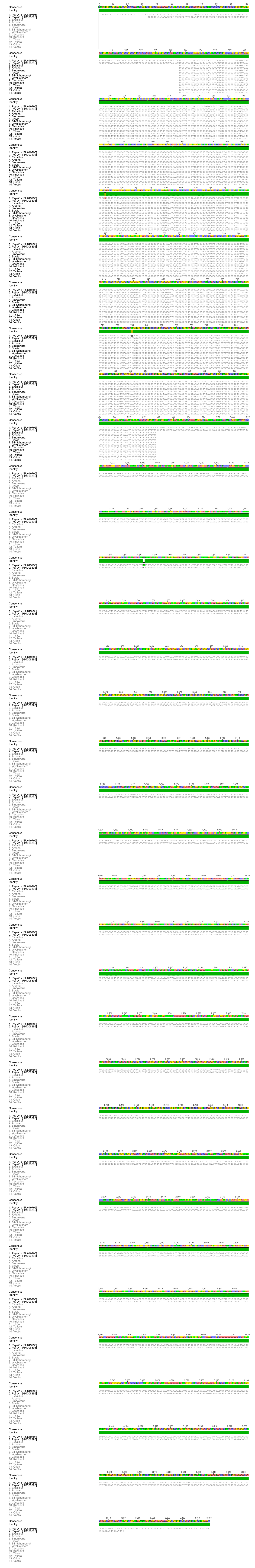

Supplement: Additional file 2 — Alignment of sequences of PSY7A5_F/R amplicons from Excalibur and 11 other cultivars to the Psy-A1t allele sequence of the breeding line WAWHT2074 [GenBank:HM006895] and the Psy-A1s allele sequence of the cultivar Schomburgk [GenBank:EU649795]. [file 1471-2229-13-230-S2.pdf]
